# Lightweight Image Super-Resolution Reconstruction Network Based on Multi-Order Information Optimization

**DOI:** 10.3390/s25175275

**Published:** 2025-08-25

**Authors:** Shengxuan Gao, Long Li, Wen Cui, He Jiang, Hongwei Ge

**Affiliations:** 1 School of Computer Science and Technology, Dalian University of Technology, Dalian 116024, China; 15652093118@mail.dlut.edu.cn; 2 School of Information and Control Engineering, China University of Mining and Technology, Xuzhou 221116, China; longli@cumt.edu.cn (L.L.); cuiwen@cumt.edu.cn (W.C.); jianghe@cumt.edu.cn (H.J.)

**Keywords:** super-resolution, lightweight, attention mechanism, information distillation, multi-order information optimization

## Abstract

Traditional information distillation networks using single-scale convolution and simple feature fusion often result in insufficient information extraction and ineffective restoration of high-frequency details. To address this problem, we propose a lightweight image super-resolution reconstruction network based on multi-order information optimization. The core of this network lies in the enhancement and refinement of high-frequency information. Our method operates through two main stages to fully exploit the high-frequency features in images while eliminating redundant information, thereby enhancing the network’s detail restoration capability. In the high-frequency information enhancement stage, we design a self-calibration high-frequency information enhancement block. This block generates calibration weights through self-calibration branches to modulate the response strength of each pixel. It then selectively enhances critical high-frequency information. Additionally, we combine an auxiliary branch and a chunked space optimization strategy to extract local details and adaptively reinforce high-frequency features. In the high-frequency information refinement stage, we propose a multi-scale high-frequency information refinement block. First, multi-scale information is captured through multiplicity sampling to enrich the feature hierarchy. Second, the high-frequency information is further refined using a multi-branch structure incorporating wavelet convolution and band convolution, enabling the extraction of diverse detailed features. Experimental results demonstrate that our network achieves an optimal balance between complexity and performance, outperforming popular lightweight networks in both quantitative metrics and visual quality.

## 1. Introduction

Image super-resolution (SR) reconstructs a low-resolution (LR) image into a high-resolution (HR) one. It has made substantial contributions to medical imaging [1,2], smart mining [3,4], and computational photography [5,6]. Traditional SR methods fall into two categories: interpolation [7] and reconstruction [8]. However, interpolation often results in blurry, distorted images, while reconstruction is computationally intensive, hindering their practical use.

In recent years, with the development of deep learning, convolutional neural networks (CNNs) [9,10] have been widely used in image super-resolution (SR). Dong et al. [11] first introduced CNNs into SR and proposed the super-resolution convolutional neural network (SRCNN), which consists of three convolutional layers. Subsequently, the same team proposed the Fast Super-Resolution Convolutional Neural Network (FSRCNN) [12], which significantly improves inference speed by incorporating deconvolution layers and a more compact network architecture. Shi et al. [13] proposed a pixel shuffling strategy and used it to construct the efficient sub-pixel convolutional neural network (ESPCN). However, SRCNN, FSRCNN, and ESPCN have shallow network layers and poor reconstruction effects. To address these limitations, Kim et al. [14] increased the number of network layers and proposed very deep convolutional networks for single image super-resolution (VDSR), which improved network performance to some extent. Lim et al. [15] proposed enhanced deep residual networks for single image super-resolution (EDSR), which constructs an even deeper network by removing the batch normalization layer, achieving excellent reconstruction results. Although VDSR and EDSR achieved good reconstruction results, their large number of network parameters and computational overhead make it difficult to deploy them on mobile devices for practical applications. Consequently, researchers have proposed recursive, pruning, and information distillation methods to develop lightweight networks. Among these, the residual feature distillation network (RFDN) [16], as a typical information distillation network, refines features layer by layer through a flexible feature distillation mechanism, ensuring a lightweight network while demonstrating efficient reconstruction capabilities.

Currently, most information distillation networks still use the RFDN’s information distillation mechanism, which completes feature extraction by stacking single-scale convolution in the distillation block, directly fuses the distilled features of different layers, and then unifies all the features. Although this approach meets the lightweight network requirements, it is prone to information singularity during feature extraction and the dilution or loss of high-frequency information due to the simple fusion mechanism. To address these issues, we propose a lightweight image super-resolution reconstruction network based on multi-order information optimization (MOION). The network optimizes image detail restoration through four streamlined stages: high-frequency enhancement, information distillation, frequency refinement, and feature fusion, effectively extracting critical features while eliminating redundancy.

The core component of MOION is the multi-order information optimization block (MOIOB), which incorporates three dual-branch self-calibrating high-frequency information enhancement block (SCHIEB). These generate wavelet-derived calibration weights to amplify critical features while suppressing noise, complemented by auxiliary branches and spatial optimization for local detail extraction. Following the enhancement stage, we set up four distillation layers. Each layer compresses the enhanced multi-channel information into a small number of key features using low-dimensional convolution. This ensures the lightweight design of network. Subsequently, we construct a multi-scale high-frequency information refinement block (MSHIRB), which further refines the distilled key features through multiplicity sampling and a multi-branch feature extraction strategy, enabling the network to capture diverse image details. Finally, we introduce an enhanced spatial attention block to weight and map the processed features, further strengthening the representation of key regions and enabling full feature fusion and utilization.

The main contributions of this paper are summarized as follows:

- We propose a self-calibrating high-frequency information enhancement block (SCHIEB). By designing an adaptive high-frequency enhancement mechanism, the network can dynamically adjust feature representation across different regions, addressing the insufficient high-frequency expression in traditional distillation networks.
- We design a multi-scale high-frequency information refinement block (MSHIRB). By using a lightweight multiplicity sampling and multi-branch feature extraction method, it fully captures the remaining multi-scale information and high-frequency details, solving the problem of limited feature diversity in traditional distillation networks.
- We propose a multi-order information optimization block (MOIOB). Compared to traditional distillation blocks, our architecture establishes a complete information optimization path, enabling better extraction of high-frequency features and removal of redundant information, thus improving detail recovery.

## 2. Related Work

### 2.1. Lightweight SR Network

Deep networks, dependent on vast parameters and computational resources, pose training challenges and hinder practical applications. This has spurred interest in lightweight networks suitable for mobile deployment. Tai et al. [17] presented the deep recursive residual network (DRRN), which uses parameter sharing and recursive learning to effectively reduce network parameters. Yu et al. [18] introduced the distillation and iterative pruning network (DIPNet), adopting pruning techniques to remove redundant connections and thus enhance network efficiency and generalization ability. Sun et al. [19] proposed the spatially-adaptive feature modulation network (SAFMN), designing spatially adaptive feature modulation mechanisms to dynamically select representative features and increase information processing speed. Lu et al. [20] developed the efficient super-resolution transformer (ESRT), mixing CNN and lightweight Transformer backbone to extract deep features efficiently at a low computational cost. Li et al. [21] designed the cross-receptive focused inference network (CFIN), which combines cross-scale aggregation blocks with cross-acceptance focusing mechanisms to eliminate redundant features and achieve a good balance between network performance and complexity.

Due to hardware constraints, super-resolution networks must balance computational efficiency with enhanced detail reconstruction. To overcome this limitation, we redesign traditional information distillation through multi-order information optimization block, establishing comprehensive informa-tion optimization path. Through this optimized architectural design, our network enhances detail recovery while maintaining low computational overhead.

### 2.2. Lightweight SR Network Based on Information Distillation

Currently, researchers propose a variety of efficient information distillation networks to meet the needs of practical applications. Hui et al. [22] first proposed the information distillation network (IDN), which first performs channel segmentation of the features, processes only some of the features, and finally aggregates them with the retained original features. This approach greatly reduces the computational complexity of the network. Subsequently, Hui et al. [23] proposed the information multi-distillation network (IMDN), which gradually extracts features by cascading distillation layers and improves the efficiency of network feature extraction. Based on the IMDN architecture, Liu et al. [16] proposed RFDN, which refines the features layer by layer through a flexible feature distillation mechanism and demonstrates efficient reconstruction capability while ensuring lightweight. Kong et al. [24] proposed a residual local feature network (RLFN), which removes the feature distillation connections and significantly accelerates the network inference. Li et al. [25] proposed a blueprint separable residual network (BSRN), which reduces the redundant computation in the feature extraction block by blueprint separable convolution and enhances the distillation feature extraction capability by combining with an efficient attention block.

However, most of these networks focus on structural simplification and computational efficiency but neglect the balance between performance and complexity. The proposed multi-order information optimization network bridges this research gap. It enhances feature extraction and captures more image details. Additionally, it improves network performance with fewer added parameters, offering a better trade-off than existing information distillation networks.

## 3. Multi-Order Information Optimization Network

### 3.1. Network Architecture

**Figure 1:**
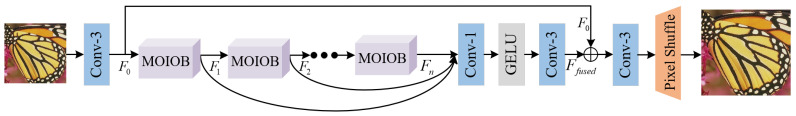
Multi-order information optimization network structure.

The overall structure of a multi-order information optimization network (MOION) is shown in Figure 1. MOION consists of four parts: shallow feature extraction, deep feature extraction, multi-layer feature fusion, and reconstruction. For the input low-resolution image $I_{LR}$, we use a 3×3 convolution to extract shallow features $F_{0}$. The process is represented as

(1) $$F_{0}=h\left(I_{LR}\right)=C_{3}\left(I_{LR}\right)$$

where h(·) denotes shallow feature extraction function and $C_{3}\left(\cdot \right)$ denotes 3×3 convolution. $F_{0}$ is fed into multiple multi-order information optimization block (MOIOB) to extract the deep features step by step, and the process is expressed as

(2) $$F_{n}=H_{\mathrm{MOIOB}}^{n}\left(H_{\mathrm{MOIOB}}^{n-1}\left(\ldots \left(H_{\mathrm{MOIOB}}^{1}\left(F_{0}\right)\right)\ldots \right)\right)$$

where $H_{\mathrm{MOIOB}}^{n}\left(\cdot \right)$ denotes the *n*th MOIOB function and $F_{n}$ denotes the output of the *n*th MOIOB. In order to fully utilize the features of all depths, they are spliced and activated by 1×1 convolutional fusion and GELU, and then the fused features are refined by 3×3 convolution, which is denoted as

(3) $$F_{fused}=C_{3}\left(C_{1}\left(Concat\left(F_{1},\ldots ,F_{n}\right)\right)\right)$$

where Concat(·) denotes feature splicing along the channel dimension, $C_{1}\left(\cdot \right)$ denotes 1×1 convolution, and $F_{fused}$ denotes fused features. In order to take advantage of residual learning, the $F_{0}$ and the fused features are summed and fed into the reconstruction part. This part consists of one 3×3 convolution and pixel shuffling operation [13] for up-sampling the image. The process is represented as

(4) $$I_{SR}=H_{rec}\left(F_{fuzed}⊕F_{0}\right)$$

where $H_{rec}\left(\cdot \right)$ denotes the reconstruction function and $I_{SR}$ denotes the output super-resolution image. In this paper, the network is trained by minimizing the $l_{1}$ loss function, which is denoted by L(θ):

(5) $$L\left(\theta \right)=\frac{1}{N}\sum_{i=1}^{N}{\left\|H_{\mathrm{MOION}}\left(I_{LR}^{i}\right)-I_{HR}^{i}\right\|}_{1}$$

where θ denotes the learnable parameters of MOION, $H_{\mathrm{MOION}}\left(\cdot \right)$ denotes the MOION function, ${∥\cdot ∥}_{1}$ denotes the number of $l_{1}$ norm, $I_{LR}^{i}$ and $I_{HR}^{i}$ denote the *i*th input LR and HR image sample pairs, *N* is the total number of samples, *i* is the number of sample serial numbers.

### 3.2. Multi-Order Information Optimization Block

**Figure 2:**
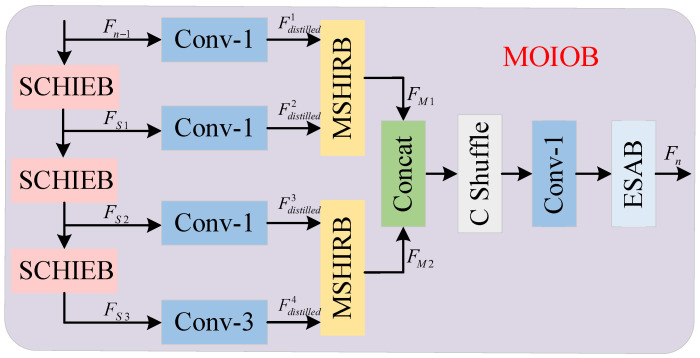
Multi-order information optimization block.

Traditional information distillation blocks typically use single-scale convolution in their backbone to extract high-frequency information and then directly fuse the distilled results. This leads to insufficiently rich and overly uniform feature information, failing to fully capture the image’s diverse details. In order to solve the above problems, we propose a multi-order information optimization block (MOIOB), which fully exploits the high-frequency information in the image through four stages: high-frequency information enhancement, information distillation, high-frequency information refinement, and information fusion. The structure of MOIOB is shown in Figure 2.

The first stage consists of three series-connected self-calibrating high-frequency information enhancement blocks (SCHIEBs) which aim to enhance the high-frequency information of the input image. Furthermore, this stage provides the subsequent information distillation and refinement stages with richer high-frequency features. Taking the first *n*th MOIOB in Figure 1 as an example, the input is $F_{n-1}$, and the high-frequency information enhancement stage can be expressed as follows:

(6) $$\begin{array}{cc} \; & F_{S1}=H_{\mathrm{SCHIEB}}\left(F_{n-1}\right),\\ \; & F_{S(i+1)}=H_{\mathrm{SCHIEB}}\left(F_{Si}\right),i=1,2 \end{array}$$

where $F_{Si}$ denotes the output of the *i*th SCHIEB and $H_{SCHIEB}^{i}\left(\cdot \right)$ denotes the SCHIEB function.

The second stage consists of three 1×1 convolution layers and one 3×3 convolution layer, which compresses the information of multiple channels into fewer key features through low-dimensional convolution to achieve information distillation. This stage intends to reduce the information redundancy, refine the feature representation via low-dimensional convolution, and ensure the lightweight of the network. The information distillation stage can be expressed as

(7) $$\begin{array}{cc} \; & F_{distilled}^{1}=H_{distill}\left(F_{n-1}\right),\\ \; & F_{distilled}^{j+1}=H_{distill}\left(F_{Si}\right),j=1,2,3 \end{array}$$

where $F_{distilled}^{i}$ and $F_{distilled}^{j+1}$ denote the distilled features and $H_{distill}\left(\cdot \right)$ denotes the information distillation function.

The third stage consists of two multi-scale high-frequency information refinement blocks (MSHIRBs) whose purpose is to refine the distilled information, further refine the high-frequency features, and optimize the final reconstruction effect. The high-frequency information refinement stage can be expressed as

(8) $$\begin{array}{cc} \; & F_{M1}=H_{\mathrm{MSHIRB}}\left(Concat\left(F_{distilled}^{1},F_{distilled}^{2}\right)\right),\\ \; & F_{M2}=H_{\mathrm{MSHIRB}}\left(Concat\left(F_{distilled}^{3},F_{distilled}^{4}\right)\right) \end{array}$$

where $F_{M1}$ and $F_{M2}$ denote the output of two MSHIRBs and $H_{MSHIRB}\left(\cdot \right)$ denotes the MSHIRB function. The outputs of the MSHIRB are fused in the fourth stage. In this paper, the features are channel rearranged using channel blending to break the isolation of the information between each channel and avoid the singularity of feature information.

Finally, the features after 1×1 convolutional smoothing are then fed into the enhanced spatial attention block (ESAB) [26] for weighted combination and feature mapping, which helps the network to focus on more discriminative features in the spatial domain to improve the efficiency of information utilization. The information fusion stage can be represented as

(9) $$F_{n}=H_{fuse}\left(Concat\left(F_{M1},F_{M2}\right)\right)$$

where $F_{n}$ denotes the output of the *n*th MOIOB and $H_{fuse}\left(\cdot \right)$ denotes the information fusion function. Through the above four stages, MOIOB can remove redundant information to optimize the information extraction and fusion process, and it improves the network’s ability to recover the details of the features.

### 3.3. Self-Calibrating High-Frequency Information Enhancement Block

In image super-resolution tasks, edges and textures are crucial for image restoration, and this information is usually embedded in the high-frequency features of the image. However, the operation of stacked convolution of conventional distillation blocks cannot dynamically adjust the feature expression in different regions, resulting in high-frequency information being easily interfered by noise, which affects the quality of image reconstruction. For this reason, we propose the self-calibrating high-frequency information enhancement block (SCHIEB) which adaptively enhances the high-frequency information through dual branching.

**Figure 3:**
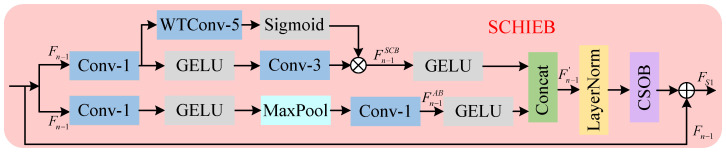
Self-calibrating high-frequency information enhancement block.

The structure of SCHIEB is shown in Figure 3, which includes self-calibrating branch (SCB) and auxiliary branch (AB). In SCB, the input features are processed in two steps: first, we use 1×1 convolution to downscale the input features to reduce the computational complexity and then extract the local high-frequency features by 3×3 convolution after activation by the GELU function; second, we introduce wavelet convolution (WTConv) [27], which realizes a larger sensory field and helps the network to capture the shape information of the image. The input features are first processed through a WTConv-5 layer and sigmoid activation to generate calibration weights. Then, they are multiplied with the outputs of a 3 × 3 convolutional layer. This operation controls pixel-wise response intensity and suppresses the noise. Finally, it can adaptively enhance the high-frequency information. Taking the first SCHIEB in Figure 2 as an example, the processing of features by SCB can be expressed as follows:

(10) $$F_{n-1}^{SCB}=\sigma \left(WTCon\nu_{5}\left(F_{n-1}\right)\right)⊙C_{3}\left(GELU\left(C_{1}\left(F_{n-1}\right)\right)\right)$$

where σ(·) denotes the sigmoid function, $WTCon\nu_{5}\left(\cdot \right)$ denotes the wavelet convolution, $F_{n-1}$ denotes the input of SCHIEB, GELU(·) denotes the GELU activation function, and $F_{n-1}^{SCB}$ denotes the output of SCB. In AB, the input features are also activated by dimensionality reduction, then the significant features in the local region are retained by maxpooling, and finally the local details are further refined by 1×1 convolution accumulation to further refine the local details to assist in enhancing the high-frequency information. The processing of features in AB can be represented as

(11) $$F_{n-1}^{AB}=C_{1}\left(MaxPool\left(GELU\left(C_{1}\left(F_{n-1}\right)\right)\right)\right)$$

where MaxPool(·) denotes maximum pooling and $F_{n-1}^{AB}\left(\cdot \right)$ denotes the output of AB.

**Figure 4:**
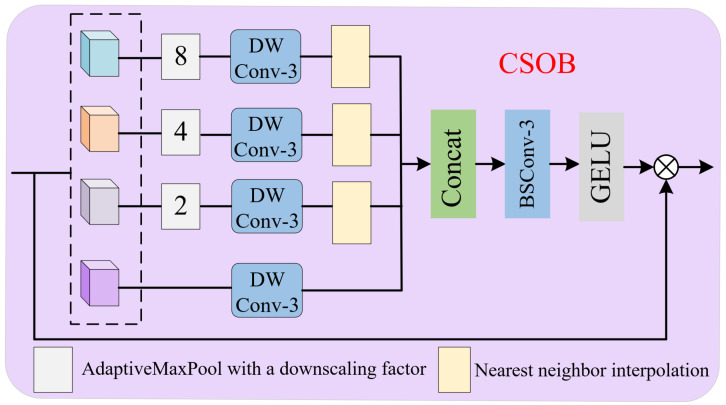
Chunked space optimization block.

The features enhanced by SCB and AB are spliced in the channel dimension after GELU activation, respectively. In order to capture more useful features in the space, we input the spliced features into the chunked space optimization block (CSOB) to further optimize the feature representation, the structure of which is shown in Figure 4. The CSOB module builds upon spatially adaptive feature modulation [19], employing feature partitioning and adaptive max-pooling for multi-scale downsampling. Local contextual information within partitioned regions is captured through depthwise convolutions, followed by upsampling and channel-wise concatenation of processed features. Spatial correlations across blocks are aggregated via efficient blueprint separable convolution [25]. The optimized features are ultimately obtained through element-wise multiplication. The CSOB effectively implements the cross-scale information interactions and further enhances the diversity of the feature expression. Finally, the CSOB-optimized features are summed with the original features through residual linkage as the output of SCHIEB. The above process can be expressed as

(12) $$\begin{array}{cc} \; & F_{n-1}^{\prime }=Concat\left(GELU\left(F_{n-1}^{SCB}\right),GELU\left(F_{n-1}^{AB}\right)\right),\\ \; & F_{S1}=F_{n-1}⊕H_{\mathrm{CSOB}}\left(LN\left(F_{n-1}^{\prime }\right)\right) \end{array}$$

where $F_{n-1}^{\prime }$ denotes the features after two-branch splicing, $F_{S1}$ denotes the output of SCHIEB, LN(·) denotes layer normalization, and $H_{\mathrm{CSOB}}\left(\cdot \right)$ denotes the CSOB function.

### 3.4. Multi-Scale High-Frequency Information Refinement Block

**Figure 5:**
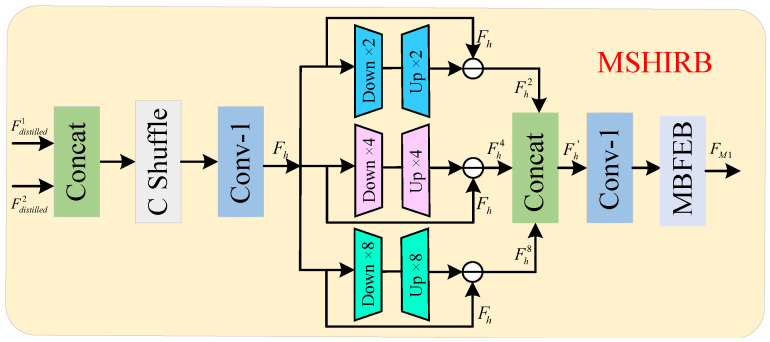
Multi-scale high-frequency information refinement block.

The traditional information distillation block directly fuses features from different layers and then unifies all the features. In this process, high-frequency information is diluted or lost due to simple weighting and fusion. In order to further refine the distillation information and retain more high-frequency features, we propose the multi-scale high-frequency information refinement block (MSHIRB) as shown in Figure 5. It takes two adjacent post-distilled features as input. First, these features are spliced along the channel dimension. Then, channel blending is applied to enhance cross-level information exchange. Finally, a 1×1 convolution reduces the dimension, lowering the network’s computational complexity.

In MSHIRB, we use multiplicity sampling to accomplish the extraction of high-frequency information. This approach can capture multi-scale high-frequency information with very little overhead. Specifically, the downsampled features are downsampled two, four, and eight times by maxpooling and recovered to the original feature map size by interpolation to obtain the features containing more low-frequency information and subtracted from the original features element by element to extract the features containing multi-scale high-frequency information. Taking the distillation features $F_{distilled}^{1}$ and $F_{distilled}^{2}$ as an example, the process of multiplicity sampling can be expressed as

(13) $$\begin{array}{cc} \; & F_{h}=C_{1}\left(H_{Cshuffle}\left(Concat\left(F_{distilled}^{1},F_{distilled}^{2}\right)\right)\right),\\ & F_{h}^{2}=F_{h}-H_{up}^{2}\left(H_{down}^{2}\left(F_{h}\right)\right),\\ & F_{h}^{4}=F_{h}-H_{up}^{4}\left(H_{down}^{4}\left(F_{h}\right)\right),\\ & F_{h}^{8}=F_{h}-H_{up}^{8}\left(H_{down}^{8}\left(F_{h}\right)\right) \end{array}$$

where $F_{h}\left(\cdot \right)$ denotes features after dimensionality reduction, $H_{Cshuffle}\left(\cdot \right)$ denotes the channel mixing operation, $F_{h}^{2}$, $F_{h}^{4}$, and $F_{h}^{8}$ are the multiscale high-frequency features obtained by multiplicity sampling, $H_{down}^{2}\left(\cdot \right)$, $H_{down}^{4}\left(\cdot \right)$ and $H_{down}^{8}\left(\cdot \right)$ indicate two-, four- and eight-time downsampling, $H_{up}^{2}\left(\cdot \right)$, $H_{up}^{4}\left(\cdot \right)$, and $H_{up}^{8}\left(\cdot \right)$ indicate two-, four-, and eight-time upsampling, respectively. The multiplicity sampling effectively retains and refines the high-frequency features in the distillation information.

**Figure 6:**
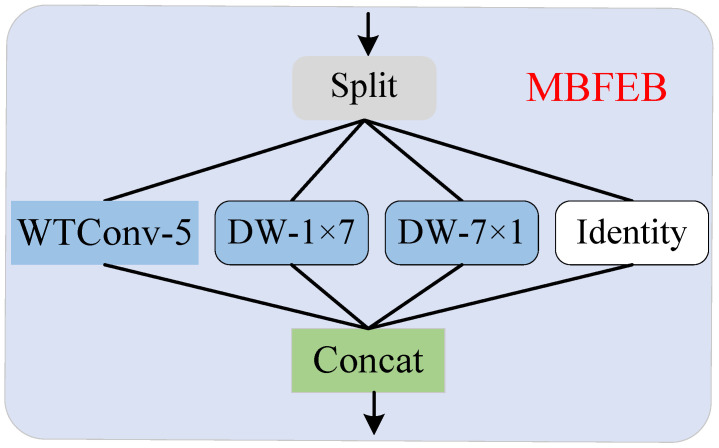
Multi-branch feature extraction block.

To further refine these features and enable the network to capture richer texture information, we propose the multi-branch feature extraction block (MBFEB) illustrated in Figure 6. The multi-scale high-frequency information, after channel splicing and dimensionality reduction, is fed into MBFEB. Within MBFEB, channel segmentation divides the information into four parts. While part of the original channel information is retained, the remaining three branches undergo wavelet and band convolutions separately. Wavelet convolution [27] captures the image’s shape information, while band convolution extracts its horizontal and vertical texture information. By fusing diverse information from different branches, MBFEB enhances the network’s ability to recover image details. The refinement process of features by MBFEB can be represented as

(14) $$\begin{array}{cc} \; & F_{h}^{\prime }=Concat\left(F_{h}^{2},F_{h}^{4},F_{h}^{8}\right),\\ \; & F_{M1}=H_{\mathrm{MBFEB}}\left(C_{1}\left(F_{h}^{\prime }\right)\right) \end{array}$$

where $F_{h}^{\prime }$ denotes the multiplicity sampled features spliced in the channel dimension, $H_{MBFEB}\left(\cdot \right)$ denotes the MBFEB function, and $F_{M1}$ denotes the output of MSHIRB.

## 4. Experimental Results and Analysis

### 4.1. Experimental Setup

The experiments are conducted in an environment with an Intel i5-13490F processor, a NVIDIA RTX 4070 graphics card, and a Pytorch 10.1 framework. The initial learning rate of the network is set to $5\times 10^{-4}$, and the number of training rounds is halved when the number of training rounds reaches 200 with a total of 1000 rounds of training. The training image cropping block size is 64×64 with 16 small blocks input per batch. The optimizer is ADAM [28] with parameters set to $\beta_{1}=0.9$, $\beta_{2}=0.999$, $\epsilon =10^{-8}$. The number of input channels to the network is 64 and the number of MOIOB is 6. All networks in the ablation experiment are trained for 300 rounds with 32 input channels and the rest of the training settings are the same as the established configuration.

### 4.2. Datasets and Evaluation Indicators

In this paper, 800 pairs of images from DIV2K [29] are used as the training set, and four publicly available datasets, Set5 [30], Set14 [31], B100 [32], and Urban100 [33], are used as the test set. The network measures the complexity by the number of parameters and floating-point operations FLOPs, and the quality of the reconstructed image is measured by peak signal to noise ratio (PSNR) and structural similarity (SSIM) [34]. The PSNR is measured in dB, and the larger the value, the higher the quality of the reconstructed image. The PSNR is calculated using the following formula:

(15) $$\begin{array}{cc} \; & MSE=\frac{1}{H\times W}\sum_{i=1}^{H}\sum_{j=1}^{W}{\left(x\left(i,j\right)-y\left(i,j\right)\right)}^{2},\\ \; & \mathrm{PSNR}=10\mathrm{lg}\left(\frac{MAX_{I}^{2}}{MSE}\right) \end{array}$$

where *x* is the reconstructed image, *y* is the real high resolution image, MSE is the mean square error, x(i,j) and y(i,j) are the pixel values at the corresponding coordinates, *H* and *W* are the height and width of the image, respectively, $MAX_{I}$ is the maximum pixel value in the image. SSIM mainly consists from the brightness, structure, and contrast to consider in the reconstruction of the quality of the image, which take a range from 0 to 1. The closer the value to 1, the higher the quality of the reconstructed image. SSIM is calculated by the following formula:

(16) $$\mathrm{SSIM}=\frac{\left(2\mu_{x}\mu_{y}+c_{1}\right)\left(2\sigma_{xy}+c_{2}\right)}{\left(\mu_{x}^{2}+\mu_{y}^{2}+c_{1}\right)\left(\sigma_{x}^{2}+\sigma_{y}^{2}+c_{2}\right)}$$

where the reconstructed image is *x*, the real high-resolution image is *y*, $\mu_{x}$ and $\mu_{y}$ are the average pixel values of *x* and *y*, respectively, $\sigma_{x}^{2}$ and $\sigma_{y}^{2}$ are the variances of *x* and *y*, respectively, $\sigma_{xy}$ is the covariance of *x* and *y*, and $C_{1}$, $C_{2}$ are constants.

### 4.3. Network Performance Comparison

#### 4.3.1. Comparison of Objective Quantitative Indicators

In order to verify the superiority of the networks proposed in this paper, MOION is compared with the current state-of-the-art lightweight networks, including EDSR-baseline [15], IMDN [23], RFDN [16], BSRN [25], SAFMN [19], DLSR [35], DRSAN [36], HAFRN [37], OSFFNet [38], and HSRNet [39]. As can be seen from Table 1, MOION achieves optimality in all metrics. As the scale factor increases, the more high-frequency information is needed for image reconstruction, the more difficult it is to reconstruct, and MOION has a more obvious advantage in large-scale reconstruction metrics compared with other networks. The Urban100 dataset, with its challenging and complex texture, can better validate the network’s reconstruction capability. Taking ×4 as an example, MOION improves PSNR by 0.27dB and SSIM by 0.0071 on Urban100 compared to HSRNet with the number of parameters greater than 1M, while the number of parameters is only 65% of HSRNet. Compared with IMDN, which has a close number of parameters, MOION improves PSNR by 0.51dB and SSIM by 0.0167 on Urban100, while the computation is 12% less than IMDN. This shows that MOION has superior performance and a good trade-off between complexity and performance.

#### 4.3.2. Comparison of Subjective Visual Effects

**Figure 7:**
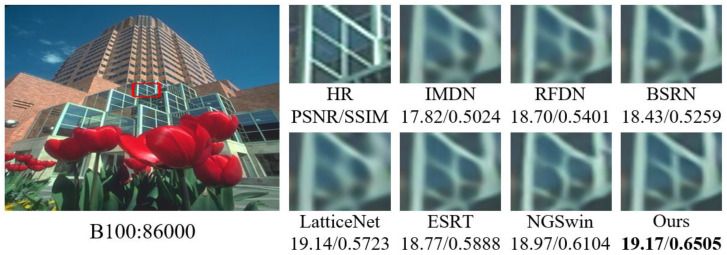
Visual comparison of different networks on B100: 86000.

**Figure 8:**
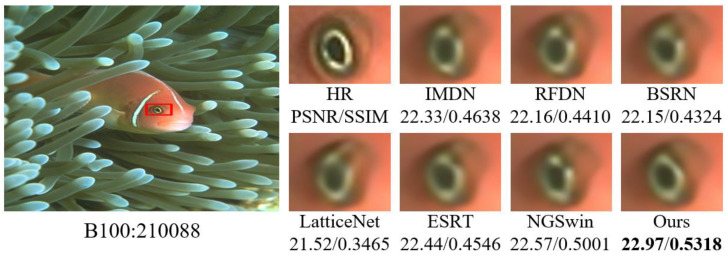
Visual comparison of different networks on B100: 210088.

**Figure 9:**
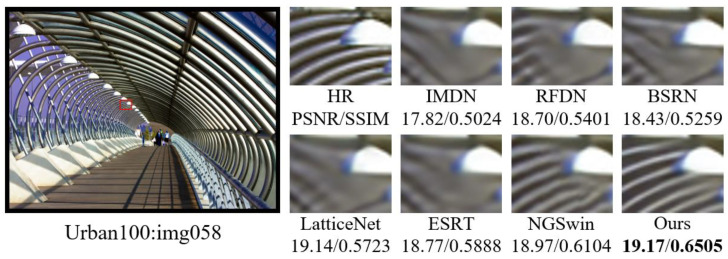
Visual comparison of different networks on Urban100: img058.

**Figure 10:**
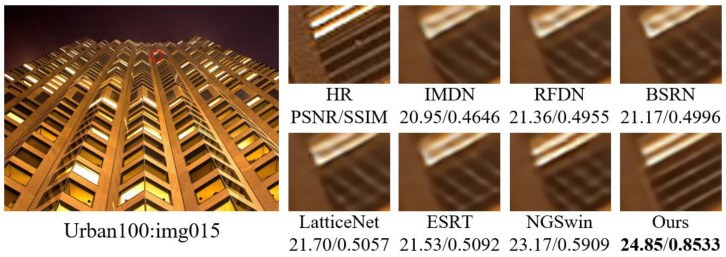
Visual comparison of different networks on Urban100: img015.

In order to visualize the reconstruction performance of MOION, images with complex texture details in B100 and Urban100 are selected for reconstruction at ×4 scale, and the reconstruction results are compared with IMDN, RFDN, BSRN, LatticeNet [43], ESRT [20], and NGSwin [44] in terms of subjective visual effects. The experimental results are shown in Figure 7, Figure 8, Figure 9 and Figure 10. In image 86000, MOION reconstructs straighter and clearer grid lines, while the rest of the networks reconstruct distorted and blurred lines. In image 210088, MOION reconstructs the fisheye shape closest to HR, while the rest of the networks reconstruct the fisheye with obvious distortion. In image 058, MOION reconstructs all curves completely, while the remaining networks fail to reconstruct them completely or are illegible. In image 015, MOION reconstructs the lines in the correct orientation, while the remaining networks all reconstruct the wrong orientation. Overall, the comparison of the reconstruction results in Figure 7, Figure 8, Figure 9 and Figure 10 further demonstrates the advanced performance of MOION.

#### 4.3.3. Comparison with Transformer-Based Networks

In recent years, the application of Transformer in SR has greatly improved the reconstruction performance and demonstrated great competitiveness compared with CNN networks. In order to further verify the superiority of MOION, seven Transformer-based lightweight networks are selected for comparison in this paper, including SwinIR-light [45], LBNet [46], ESRT, NGSwin, DRSAN [36], CFIN [21], and HCFormer [47], and their results are shown in Table 2. Compared with HCFormer, MOION achieves 16 optimal and 6 suboptimal out of 24 metrics, while HCFormer achieves 7 optimal and 5 suboptimal. The total number of optimal and suboptimal values of MOION is more than that of HCFormer, and the number of parameters required for the network at ×2, ×3, and ×4 scales is reduced by 10.4%, 10.6%, and 11% respectively. Compared with SwinIR-light, the total number of most and second best of MOION is still more than SwinIR-light, and the number of parameters and computation required by the network is lower than that of SwinIR-light in all scales. Compared with the rest of the networks, MOION achieves the optimum in most of the metrics, which further validates the superiority of MOION in image reconstruction.

### 4.4. Ablation Studies

In order to investigate the effect of the main blocks of the network on the performance, we conduct ablation experiments on WTConv-5, which provides self-calibrating weights, CSOB, a chunked spatial optimization block, and MSHIRB, a multiscale high-frequency information refinement block, respectively. The test dataset is Urban100 with complex texture and a scale factor of ×4. The network with the three blocks removed is used as the baseline, and each block is added to the baseline for reconstruction, respectively, and the results are shown in Table 3. From the results, it can be seen that when only WTConv-5 is used; compared to the baseline, PSNR is improved by 0.12 dB, SSIM is improved by 0.0036, while the number of parameters and computation are only increased by 38 K and 0.85 G, respectively. When only CSOB and MSHIRB are used, the network also obtains a large performance improvement at the cost of a small increase in overhead. This proves the effectiveness of each block. When only any two blocks are used, the network obtains a greater performance improvement compared to using each block individually, and when all three blocks are used simultaneously, the PSNR improves by 0.21 dB compared to the baseline. The SSIM improves by 0.0076, optimizing the performance with only a small increase in the network complexity. This demonstrates the synergistic effects between the blocks.

**Figure 11:**
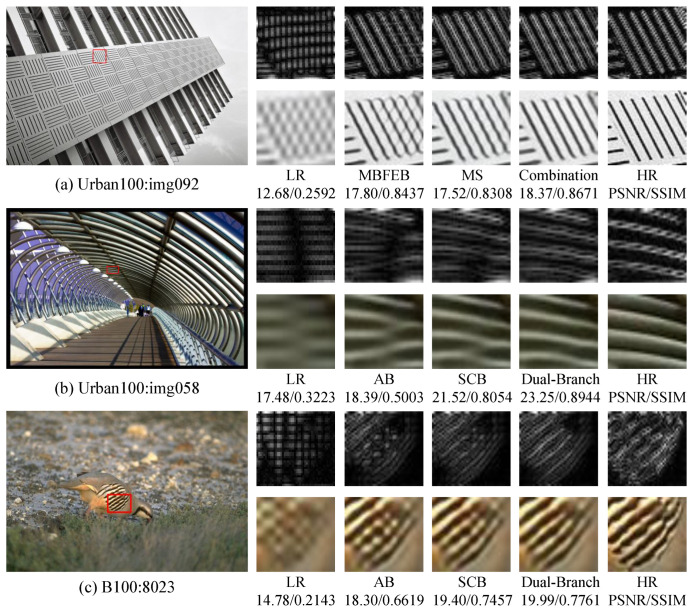
Reconstructed images and their high-frequency component images.

MSHIRB mainly consists of multiplicity sampling and multi-branch feature extraction block MBFEB. In order to investigate the effect of the two blocks on the network performance, the removal of the two blocks is used as the baseline, and the experiments are conducted by adding each block to the baseline, respectively, and the results are shown in Table 4. When only multiplicity sampling is used, the network improves by 0.02 dB compared to the baseline PSNR and 0.0008 for SSIM. When only MBFEB is used, the network improves by 0.07 dB compared to the baseline PSNR and 0.0023 for SSIM. When both are used at the same time, the performance is optimized. To visually demonstrate the effects of multiplicity sampling and MBFEB on image reconstruction, we conducted image restoration using three network configurations: (1) employing only multiplicity sampling, (2) utilizing solely MBFEB, and (3) integrating both blocks simultaneously. The reconstructed images and their corresponding high-frequency information are shown in Figure 11a. As demonstrated, both MBFEB and multiplicity sampling contribute to capturing more high-frequency components. However, when used individually, each block still leaves some artifacts in the reconstructed images. The combined use of both blocks enables more accurate high-frequency information recovery, consequently yielding optimal reconstruction quality. This demonstrates the complementary nature of multiplicity sampling for high-frequency information and the ability of MBFEB to refine the features, while their synergistic action is required to better accomplish the function of high-frequency information refinement.

To validate the rationale of SCHIEB’s dual-branch architecture, we conducted ablation studies by retaining only the self-calibrating branch (SCB) or auxiliary branch (AB) individually. As shown in Table 5, the dual-branch configuration achieves optimal performance, with SCB outperforming AB in single-branch tests. The SCB uses wavelet convolution to capture large-scale pixel relationships. It generates calibration weights that dynamically adjust regional feature representations, emphasizing high-frequency components and significantly enhancing high-frequency information. In contrast, the AB primarily preserves local salient features through max-pooling operations but lacks self-calibrating attention mechanisms for high-frequency compensation. To visually compare the effects of each branch, we performed image reconstruction using individual branches (SCB or AB) and dual branches. The reconstructed images and their high-frequency components are shown in Figure 11b,c. It can be observed that SCB helps the network reconstruct more high-frequency components, while AB compensates for partial high-frequency information. The dual-branch configuration achieves the highest reconstruction quality. This structural comparison shows that the dual-branch design combines their complementary advantages synergistically. It adaptively enhances high-frequency information and improves the ability to recover detailed textures.

To evaluate the impact of convolution kernel sizes in MBFEB, we tested six configurations combining wavelet convolution (WTConv) and dual strip convolutions (DW), where “5-7-7” denotes WTConv-5, DW-1×7, and DW-7×1. As shown in Table 6, larger kernels in both wavelet and strip convolutions consistently enhance network performance while maintaining moderate parameter and computational costs. Specifically, expanding the wavelet convolution kernel improves the capture of broad shape features, while larger strip convolution kernels strengthen modeling of long-range horizontal/vertical texture dependencies. Notably, since each MBFEB branch processes only a subset of channel features, the 5–7–7 configuration achieves optimal performance with minimal computational overhead.

## 5. Conclusions

This paper proposes a lightweight image super-resolution reconstruction network based on multi-order information optimization. The core of the network lies in the enhancement and refinement of high-frequency information. Through multiple stages, it fully extracts high-frequency features and removes redundant information to improve detail restoration. For high-frequency enhancement, we design a SCHIEB that regulates pixel-wise response intensities through learnable calibration weights. This block incorporates an auxiliary branch with chunked space optimization to adaptively enhance high-frequency components while preserving local structural details. In the refinement stage, we propose an MSHIRB. It first captures multi-scale information via multiplicity sampling, and then uses a multi-branch structure with wavelet and band convolutions to extract diverse detail features, further refining high-frequency information. Together, these blocks address the limitations of traditional distillation networks in high-frequency recovery and detail reconstruction. Experimental results show that the proposed network achieves competitive quantitative metrics and visual reconstruction quality while maintaining good balance between complexity and performance.

## Figures and Tables

**Table 1:** Comparison of metrics under the baseline dataset when the scale factor is ×2, ×3, and ×4. **Bold** is optimal, underlined is sub-optimal, and - indicates that the network was not tested for this condition.

Scale	Method	Params	FLOPs	Set5	Set14	B100	Urban100
				PSNR/SSIM	PSNR/SSIM	PSNR/SSIM	PSNR/SSIM
×2	EDSR-baseline [15]	1370 K	316.3 G	37.99/0.9604	33.57/0.9175	32.16/0.8994	31.98/0.9272
	IMDN [23]	694 K	186.7 G	38.00/0.9605	33.63/0.9177	32.19/0.8996	32.17/0.9283
	RFDN [16]	534 K	95.0 G	38.05/0.9606	33.68/0.9184	32.16/0.8994	32.12/0.9278
	BSRN [25]	332 K	73.0 G	38.10/0.9610	33.74/0.9193	32.24/0.9006	32.34/0.9303
	SAFMN [19]	228 K	52.0 G	38.00/0.9605	33.54/0.9177	32.16/0.8995	31.84/0.9256
	DLSR [35]	322 K	68.0 G	38.04/0.9606	33.67/0.9183	32.21/0.9002	32.26/0.9297
	DRSAN [36]	1190 K	274.6 G	38.14/**0.9611**	33.75/0.9188	32.25/0.9010	32.46/0.9317
	HAFRN [37]	496 K	-	38.05/0.9606	33.66/0.9187	32.21/0.8999	32.20/0.9289
	OSFFNet [38]	516 K	83.2 G	38.11/0.9610	33.72/0.9190	32.29/0.9012	32.67/0.9331
	HSRNet [39]	1260 K	-	38.07/0.9607	33.78/0.9197	32.26/0.9006	32.53/0.9320
	DWCAN [40]	401 K	-	37.60/0.9598	33.33/0.9160	32.07/0.8987	31.95/0.9267
	MSWSR [41]	312 K	243.3 G	38.01/0.9610	33.71/0.9193	32.22/0.9003	32.29/0.9301
	SRConvNet-L [42]	885 K	160 G	38.14/0.9610	33.81/0.9199	32.28/0.9010	32.59/0.9321
	MOION	816 K	163.74 G	**38.16**/**0.9611**	**33.92**/**0.9204**	**32.32**/**0.9014**	**32.69**/**0.9339**
×3	EDSR-baseline [15]	1555 K	160.2 G	34.37/0.9270	30.28/0.8417	29.09/0.8052	28.15/0.8527
	IMDN [23]	703 K	84.0 G	34.36/0.9270	30.32/0.8417	29.09/0.8046	28.17/0.8519
	RFDN [16]	541 K	42.2 G	34.41/0.9273	30.34/0.8420	29.09/0.8050	28.21/0.8525
	BSRN [25]	340 K	33.3 G	34.46/0.9277	30.47/0.8449	29.18/0.8068	28.39/0.8567
	SAFMN [19]	233 K	23.0 G	34.34/0.9267	30.33/0.8418	29.08/0.8048	27.95/0.8474
	DLSR [35]	329 K	-	34.49/0.9279	30.39/0.8428	29.13/0.8061	28.26/0.8548
	DRSAN [36]	1290 K	133.4 G	34.59/0.9286	30.42/0.8443	29.18/0.8079	28.52/0.8593
	HAFRN [37]	505 K	-	34.45/0.9276	30.40/0.8433	29.12/0.8058	28.16/0.8528
	OSFFNet [38]	524 K	37.8 G	34.58/0.9287	30.48/0.8450	29.21/0.8080	28.49/0.8595
	HSRNet [39]	-	-	34.47/0.9278	30.40/0.8435	29.15/0.8066	28.42/0.8579
	DWCAN [40]	401 K	-	34.29/0.9258	30.29/0.8410	29.00/0.8027	28.18/0.8521
	MSWSR [41]	307 K	249.6 G	34.40/0.9277	30.35/0.8437	29.12/0.8067	28.22/0.8548
	SRConvNet-L [42]	906 K	74 G	34.59/0.9288	30.50/0.8455	29.22/0.8081	28.56/0.8600
	MOION	825 K	73.72 G	**34.69**/**0.9294**	**30.57**/**0.8467**	**29.24**/**0.8091**	**28.68**/**0.8629**
×4	EDSR-baseline [15]	1518 K	114.0 G	32.09/0.8938	28.58/0.7813	27.57/0.7357	26.04/0.7849
	IMDN [23]	715 K	48.0 G	32.21/0.8948	28.58/0.7811	27.56/0.7353	26.04/0.7838
	RFDN [16]	550 K	23.9 G	32.24/0.8952	28.61/0.7819	27.57/0.7360	26.11/0.7858
	BSRN [25]	352 K	19.4 G	32.35/0.8966	28.73/0.7847	27.65/0.7387	26.27/0.7908
	SAFMN [19]	240 K	14.0 G	32.18/0.8948	28.60/0.7813	27.58/0.7359	25.97/0.7809
	DLSR [35]	338 K	20 G	32.33/0.8963	28.68/0.7832	27.61/0.7374	26.19/0.7892
	DRSAN [36]	1270 K	88.7 G	32.34/0.8960	28.65/0.7841	27.63/0.7390	26.33/0.7936
	HAFRN [37]	517 K	-	32.24/0.8953	28.60/0.7816	27.58/0.7365	26.02/0.7849
	OSFFNet [38]	537 K	22.0 G	32.39/0.8976	28.75/0.7852	27.66/0.7393	26.36/0.7950
	HSRNet [39]	1285 K	-	32.28/0.8960	28.68/0.7840	27.64/0.7388	26.28/0.7934
	DWCAN [40]	401 K	-	32.20/0.8938	28.56/0.2809	27.41/0.7339	26.06/0.7851
	MSWSR [41]	316 K	257.6 G	32.26/0.8966	28.67/0.7843	27.62/0.7379	26.17/0.7896
	SRConvNet-L [42]	902 K	45 G	32.44/0.8976	28.77/0.7857	27.69/0.7402	26.47/0.7970
	MOION	837 K	42.13 G	**32.51**/**0.8984**	**28.85**/**0.7874**	**27.72**/**0.7418**	**26.55**/**0.8005**

**Table 2:** Comparison with Transformer network metrics for scale factors of ×2, ×3, and ×4. **Bold** is optimal, underlined is sub-optimal, and - indicates that the network was not tested for this condition.

Scale	Method	Params	FLOPs	Set5	Set14	B100	Urban100
				PSNR/SSIM	PSNR/SSIM	PSNR/SSIM	PSNR/SSIM
×2	SwinIR-light [45]	878 K	195.6 G	38.14/**0.9611**	33.86/0.9206	32.31/0.9012	**32.76**/0.9340
	LBNet [46]	-	-	-	-	-	-
	ESRT [20]	677 K	191.4 G	38.03/0.9600	33.75/0.9184	32.25/0.9001	32.58/0.9318
	NGSwin [44]	998 K	140.4 G	38.05/0.9610	33.79/0.9199	32.27/0.9008	32.53/0.9324
	DRSAN [36]	1190 K	274.6 G	38.14/**0.9611**	33.75/0.9188	32.25/0.9010	32.46/0.9317
	CFIN [21]	675 K	116.9 G	38.14/0.9610	33.80/0.9199	32.26/0.9006	32.48/0.9311
	HCFormer [47]	911 K	-	38.06/0.9609	34.18/**0.9253**	**32.45**/**0.9051**	32.67/**0.9359**
	MOION	816 K	163.74 G	**38.16**/**0.9611**	**33.92**/0.9204	32.32 **/** 0.9014	32.69/0.9339
×3	SwinIR-light [45]	886 K	87.2 G	34.62/0.9289	30.54/0.8463	29.20/0.8082	28.66/0.8624
	LBNet [46]	736 K	68.4 G	34.47/0.9277	30.38/0.8417	29.13/0.8061	28.42/0.8559
	ESRT [20]	770 K	96.4 G	34.42/0.9268	30.43/0.8433	29.15/0.8063	28.46/0.8574
	NGSwin [44]	1007 K	66.6 G	34.52/0.9282	30.53/0.8456	29.19/0.8078	28.52/0.8603
	DRSAN [36]	1290 K	133.4 G	34.59/0.9286	30.42/0.8443	29.18/0.8079	28.52/0.8593
	CFIN [21]	681 K	53.5 G	34.65/0.9289	30.45/0.8443	29.18/0.8071	28.49/0.8583
	HCFormer [47]	923 K	-	34.51/0.9279	30.55/0.8459	**29.31**/**0.8104**	28.56/0.8613
	MOION	825 K	73.72 G	**34.69**/**0.9294**	**30.57**/**0.8467**	29.24/0.8091	**28.68**/**0.8629**
×4	SwinIR-light [45]	897 K	49.6 G	32.44/0.8976	28.77/0.7858	27.69/0.7406	26.47/0.7980
	LBNet [46]	742 K	38.9 G	32.29/0.8960	28.68/0.7832	27.62/0.7382	26.27/0.7906
	ESRT [20]	751 K	67.7 G	32.19/0.8947	28.69/0.7833	27.69/0.7379	26.39/0.7962
	NGSwin [44]	1019 K	36.4 G	32.33/0.8963	28.78/0.7859	27.66/0.7396	26.45/0.7963
	DRSAN [36]	1270 K	88.7 G	32.34/0.8960	28.65/0.7841	27.63/0.7390	26.33/0.7936
	CFIN [21]	699 K	31.2 G	32.49/**0.8985**	28.74/0.7849	27.68/0.7396	26.39/0.7946
	HCFormer [47]	940 K	58.7 G	32.41/0.8976	28.84/**0.7874**	27.66/0.7413	26.51/0.7987
	MOION	837 K	42.13 G	**32.51**/0.8984	**28.85**/**0.7874**	**27.72**/**0.7418**	**26.55**/**0.8005**

**Table 3:** Impact of different modules on network performance. **Bold** is optimal, × means adding this block, ✔ means removing this block.

Scale	WTConv-5	CSOB	MSHIRB	Params	FLOPs	Urban100
						PSNR/SSIM
×4	×	×	×	162 K	8.79 G	25.73/0.7734
	✔	×	×	200 K	9.64 G	25.85/0.7770
	×	✔	×	194 K	10.30 G	25.84/0.7771
	×	×	✔	175 K	9.43 G	25.82/0.7758
	✔	✔	×	231 K	11.14 G	25.90/0.7797
	×	✔	✔	207 K	10.93 G	25.92/0.7806
	✔	×	✔	213 K	10.27 G	25.88/0.7789
	✔	✔	✔	244 K	11.78 G	**25.94**/**0.7810**

**Table 4:** Effect of multiplicity sampling and multi-branch feature extraction on performance. **Bold** is optimal, × means adding this block, ✔ means removing this block.

Scale	Multiplicity Sampling (MS)	MBFEB	Params	FLOPs	Urban100
					PSNR/SSIM
×4	×	×	162 K	8.79 G	25.73/0.7734
	×	✔	166 K	8.89 G	25.80/0.7757
	✔	×	172 K	9.34 G	25.75/0.7742
	✔	✔	175 K	9.43 G	**25.82**/**0.7758**

**Table 5:** The impact of dual-branch on performance in SCHIEB. **Bold** is optimal.

Scale	Branch Name	Params	FLOPs	Urban100
				PSNR/SSIM
×4	SCB	195 K	9.21 G	25.81/0.7765
	AB	121 K	6.39 G	25.61/0.7692
	Dual-Branch	200 K	9.64 G	**25.85**/**0.7770**

**Table 6:** The influence of different convolutional kernel sizes on performance in MBFEB. **Bold** is optimal.

Scale	Combination	Params	FLOPs	Urban100
				PSNR/SSIM
×4	3-3-3	164 K	8.83 G	25.70/0.7724
	3-5-5	164 K	8.84 G	25.72/0.7725
	3-7-7	164 K	8.84 G	25.73/0.7733
	5-3-3	166 K	8.87 G	25.75/0.7735
	5-5-5	166 K	8.88 G	25.77/0.7747
	5-7-7	166 K	8.89 G	**25.80**/**0.7757**

## Data Availability

The raw data supporting the conclusions of this article will be made available by the authors on request.
